# Supplementary material for: Exploratory Study on COPD Phenotypes and their Progression: Integrating SPECT and qCT Imaging Analysis
Source: medRxiv. 2024 Apr 13:2024.04.10.24305577. Preprint. [Version 1] doi: 10.1101/2024.04.10.24305577 (PMC11030493; doi:10.1101/2024.04.10.24305577)
Supplement: Supplement 1 [file media-1.pdf]

SUPPLEMENTARY MATERIAL

Table S1. Demographic data and PFT results of all the subjects at three visits. R_{aw} is the airway resistance measured by plethysmography.

**V0**												
	**Gender**	**Age (yrs.)**	**BMI**	**Wight (kg)**	**Height (cm)**	**FEV1% predicted**	**FEV1/FVC**	**GOLD Stage**	**TLC (L)**	**FRC (L)**	**RV (L)**	**R_{aw} (cmH2O/L/Sec)**
Subj. 1	male	71-80	31.6	108.0	185	73	53	2	7.48	5.03	2.98	1.18
Subj. 2	male	81-90	22.9	65.5	169	50	28	2	7.91	6.11	3.88	1.31
Subj. 3	male	51-60	25.6	88.6	186	63	51	2	7.21	3.83	2.24	1.04
Subj. 4	male	51-60	21.4	61.1	169	73	56	2	6.93	4.73	2.81	1.27
Subj. 5	male	51-60	31.6	85.0	164	36	28	3	6.79	4.79	3.24	1.75
Subj. 6	male	51-60	29.3	92.8	178	83	59	1	6.76	3.71	1.22	1.26
Subj. 7	female	51-60	27.0	75.4	167	77	71	0	5.07	2.63	1.99	1.34
Subj. 8	female	61-70	35.5	91.0	160	88	77	0	4.23	1.67	1.53	1.76
**Mean±SD**			28.1 ± 4.5	83.4 ± 14.4	172.3 ± 9.0	67.9 ± 16.3	52.9 ± 16.6		6.5 ± 1.2	4.1 ± 1.3	2.5 ± 0.8	1.4 ± 0.2
**V1**												
	**Gender**	**Age (yrs.)**	**BMI**	**Wight (kg)**	**Height (cm)**	**FEV1% predicted**	**FEV1/FVC**	**GOLD Stage**	**TLC (L)**	**FRC (L)**	**RV (L)**	**R_{aw} (cmH2O/L/Sec)**
Subj. 1	male		28.7	98.2	185	74	56	2	7.76	5.71	3.54	1.12
Subj. 2	male		26.0	74.3	169	39	26	3	7.76	6.02	4.4	1.4
Subj. 3	male		26.2	90.5	186	61	51	2	7.62	3.7	2.75	1.04
Subj. 4	male		20.5	58.6	169	68	58	2	7.2	4.71	3.12	1.25
Subj. 5	male		30.6	82.4	164	31	25	3	7.1	4.94	3.73	1.71
Subj. 6	male		29.3	92.8	178	78	58	2	7.37	3.71	2.06	1.26
Subj. 7	female		26.8	75.7	168	75	71	0	4.69	2.84	1.68	1.34
Subj. 8	female		33.6	86	160	97	78	0	4.23	2.21	1.34	1.69
**Mean±SD**			27.7 ± 3.6	172.4 ± 9.0	82.3 ± 11.8	65.4 ± 20.1	52.9 ± 17.8		6.7 ± 1.3	4.2 ± 1.3	2.8 ± 1.0	1.4 ± 0.2
**V2**												
	**Gender**	**Age (yrs.)**	**BMI**	**Wight (kg)**	**Height (cm)**	**FEV1% predicted**	**FEV1/FVC**	**GOLD Stage**	**TLC (L)**	**FRC (L)**	**RV (L)**	**R_{aw} (cmH2O/L/Sec)**
Subj. 1	male		28.7	98.2	185	76	53	2	7.6	5.29	3.09	1.12
Subj. 2	male		26.0	74.3	169	51	29	2	8.11	6.31	4.66	1.4
Subj. 3	male		25.4	87.8	186	62	51	2	7.6	3.82	2.68	1.06
Subj. 4	male		20.8	59.3	169	78	60	2	7.05	4.61	3.26	1.24
Subj. 5	male		30.6	82.4	164	33	28	3	7.17	5.21	3.76	1.71
Subj. 6	male		29.3	92.8	178	81	59	1	6.89	3.07	1.63	NA

Subj. 7	female		27.0	75.4	167	73	71	0	4.98	2.7	2.1	1.31
Subj. 8	female		33.6	86	160	97	78	0	4.33	1.93	1.49	NA
**Mean±SD**			27.7 ± 3.6	172.3 ± 9.0	82.0 ± 11.4	68.9 ± 18.5	53.6 ± 16.7		6.7 ± 1.3	4.1 ± 1.4	2.8 ± 1.0	1.3 ± 0.2

Table S2. TC% and ΔV_{air}^{F} of each lobe at each visit.

**ID**	**TC%**	**Lobe**	**Visit**	**ΔV_{air}^{F}**
Subj.3	0.192	LLL	V0	0.154
Subj.4	0.281	LLL	V0	0.296
Subj.5	0.183	LLL	V0	0.149
Subj.6	0.229	LLL	V0	0.153
Subj.7	0.284	LLL	V0	0.215
Subj.8	0.295	LLL	V0	0.227
Subj.3	0.272	LUL	V0	0.291
Subj.4	0.215	LUL	V0	0.200
Subj.5	0.316	LUL	V0	0.336
Subj.6	0.258	LUL	V0	0.273
Subj.7	0.183	LUL	V0	0.217
Subj.8	0.186	LUL	V0	0.221
Subj.3	0.180	RLL	V0	0.165
Subj.4	0.283	RLL	V0	0.268
Subj.5	0.191	RLL	V0	0.172
Subj.6	0.237	RLL	V0	0.035
Subj.7	0.291	RLL	V0	0.223
Subj.8	0.290	RLL	V0	0.237
Subj.3	0.037	RML	V0	0.035
Subj.4	0.059	RML	V0	0.066
Subj.5	0.075	RML	V0	0.107
Subj.6	0.055	RML	V0	0.427
Subj.7	0.049	RML	V0	0.064
Subj.8	0.062	RML	V0	0.084
Subj.3	0.319	RUL	V0	0.355
Subj.4	0.163	RUL	V0	0.170
Subj.5	0.234	RUL	V0	0.236
Subj.6	0.222	RUL	V0	0.112
Subj.7	0.193	RUL	V0	0.281
Subj.8	0.167	RUL	V0	0.230
Subj.1	0.242	LUL	V1	0.178
Subj.2	0.287	LUL	V1	0.305
Subj.3	0.311	LUL	V1	0.283
Subj.4	0.193	LUL	V1	0.212
Subj.5	0.330	LUL	V1	0.311
Subj.6	0.259	LUL	V1	0.258
Subj.7	0.183	LUL	V1	0.186
Subj.8	0.250	LUL	V1	0.180
Subj.1	0.151	LLL	V1	0.281
Subj.2	0.170	LLL	V1	0.119
Subj.3	0.126	LLL	V1	0.178
Subj.4	0.345	LLL	V1	0.268
Subj.5	0.144	LLL	V1	0.176

Subj.6	0.185	LLL	V1	0.223
Subj.7	0.244	LLL	V1	0.287
Subj.8	0.182	LLL	V1	0.297
Subj.1	0.203	RUL	V1	0.139
Subj.2	0.306	RUL	V1	0.322
Subj.3	0.417	RUL	V1	0.331
Subj.4	0.169	RUL	V1	0.173
Subj.5	0.211	RUL	V1	0.254
Subj.6	0.258	RUL	V1	0.200
Subj.7	0.237	RUL	V1	0.187
Subj.8	0.258	RUL	V1	0.170
Subj.1	0.138	RML	V1	0.109
Subj.2	0.110	RML	V1	0.121
Subj.3	0.026	RML	V1	0.038
Subj.4	0.053	RML	V1	0.064
Subj.5	0.103	RML	V1	0.073
Subj.6	0.084	RML	V1	0.095
Subj.7	0.054	RML	V1	0.049
Subj.8	0.086	RML	V1	0.063
Subj.1	0.266	RLL	V1	0.294
Subj.2	0.128	RLL	V1	0.133
Subj.3	0.121	RLL	V1	0.171
Subj.4	0.240	RLL	V1	0.285
Subj.5	0.212	RLL	V1	0.186
Subj.6	0.215	RLL	V1	0.224
Subj.7	0.282	RLL	V1	0.292
Subj.8	0.225	RLL	V1	0.291
Subj.1	0.271	LUL	V2	0.192
Subj.2	0.273	LUL	V2	0.337
Subj.3	0.365	LUL	V2	0.312
Subj.4	0.209	LUL	V2	0.235
Subj.5	0.343	LUL	V2	0.300
Subj.6	0.286	LUL	V2	0.259
Subj.7	0.205	LUL	V2	0.202
Subj.8	0.246	LUL	V2	0.195
Subj.1	0.127	LLL	V2	0.267
Subj.2	0.193	LLL	V2	0.095
Subj.3	0.113	LLL	V2	0.109
Subj.4	0.372	LLL	V2	0.245
Subj.5	0.153	LLL	V2	0.196
Subj.6	0.130	LLL	V2	0.221
Subj.7	0.231	LLL	V2	0.260
Subj.8	0.194	LLL	V2	0.283
Subj.1	0.224	RUL	V2	0.157
Subj.2	0.279	RUL	V2	0.319
Subj.3	0.401	RUL	V2	0.411

Subj.4	0.151	RUL	V2	0.179
Subj.5	0.220	RUL	V2	0.246
Subj.6	0.293	RUL	V2	0.241
Subj.7	0.267	RUL	V2	0.210
Subj.8	0.235	RUL	V2	0.163
Subj.1	0.161	RML	V2	0.112
Subj.2	0.113	RML	V2	0.124
Subj.3	0.020	RML	V2	0.034
Subj.4	0.047	RML	V2	0.063
Subj.5	0.090	RML	V2	0.066
Subj.6	0.121	RML	V2	0.069
Subj.7	0.058	RML	V2	0.057
Subj.8	0.093	RML	V2	0.055
Subj.1	0.217	RLL	V2	0.271
Subj.2	0.141	RLL	V2	0.124
Subj.3	0.100	RLL	V2	0.134
Subj.4	0.220	RLL	V2	0.279
Subj.5	0.194	RLL	V2	0.192
Subj.6	0.169	RLL	V2	0.211
Subj.7	0.238	RLL	V2	0.271
Subj.8	0.233	RLL	V2	0.304

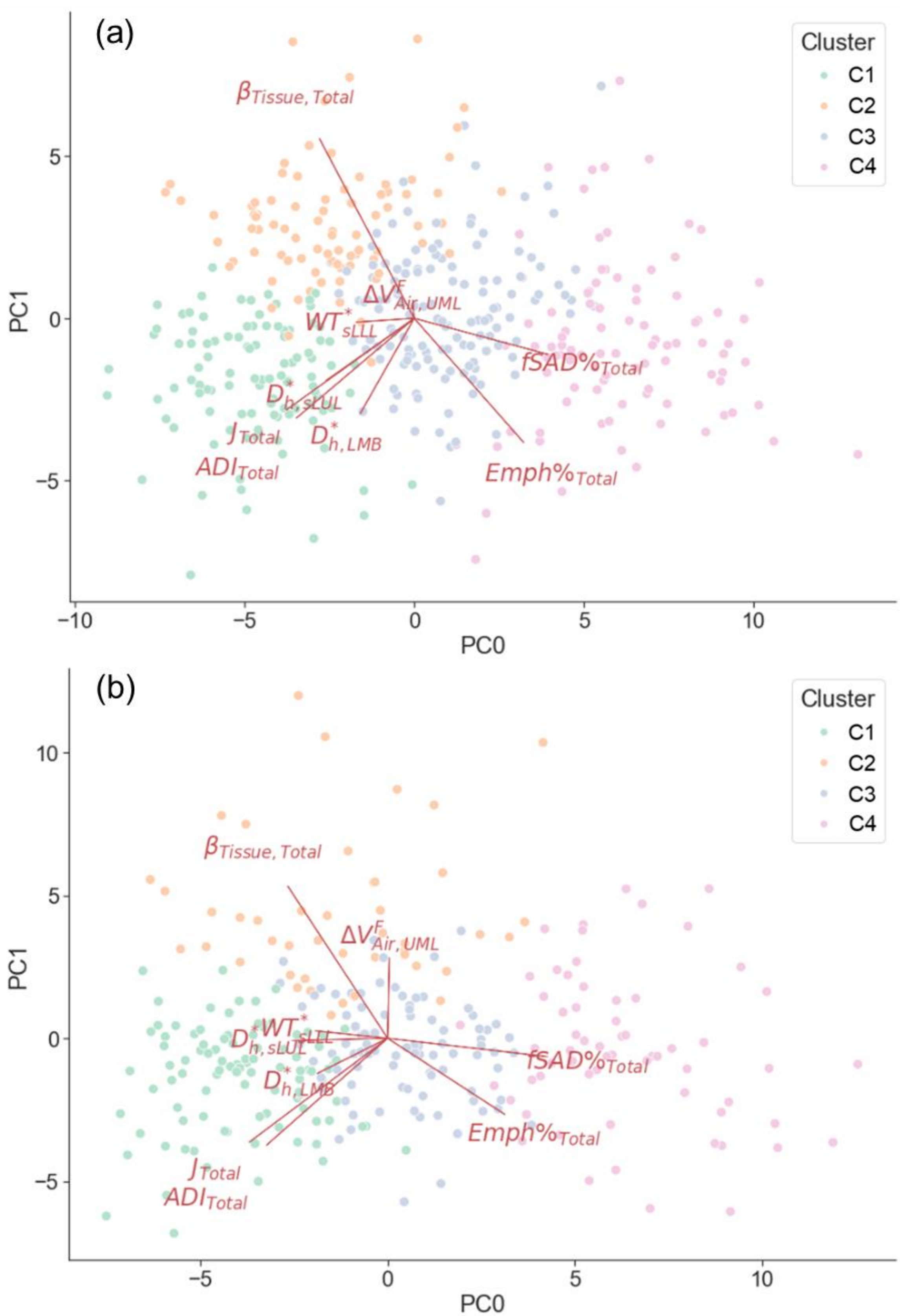

Figure S1. Biplots of the (a) former and (b) current smoker's data. The scattered points were the PC scores of observations and the arrows demonstrated the contributions and directions of the selected imaging metrics to the PC0 and PC1.

Supplementary Content 1: Multiscale qCT Biomarkers

The multiscale qCT biomarkers included lung structural and functional variables. The structural variables, describing the regional alterations of lung structures, included bifurcation angles (θ) between the children branches of trachea and right main bronchus (RMB), airway circularity (Cr), normalized airway wall thickness (WT^*), and normalized airway hydraulic diameter (D_h^*). Decreased θ and decreased Cr were found to be associated with airflow limitation (1) and increased functional small airway disease (2), respectively. The dimensions of wall thickness and hydraulic diameter were normalized by predicted trachea wall thickness and hydraulic diameter from healthy controls to eliminate inter-subject variability due to sex, age, and height (3). WT^* and D_h^* reveal the effects of wall thickening and luminal narrowing on airway obstructions caused by inflammation and hyper-responsiveness, respectively. The functional variables, capturing the regional alterations of lung functions, included fractional air volume change (ΔV_{air}^{F}), determinant of Jacobian matrix (J), anisotropic deformation index (ADI), fraction-based small airways disease (fSAD%), fraction-based emphysema (Emph%), and tissue fraction at TLC (β_{tissue}). ΔV_{air}^{F} was quantified by the ratio of the lobar air-volume change to the whole lung air-volume change. The ratio of the air-volume change of the upper lobes to the air-volume change of the middle and lower lobes combined ($\Delta V_{air,UML}^{F}$) was also calculated. Jacobian (J) is a measure of local specific volume assessing the functional capacity of lung tissue. ADI is a measure of the magnitude of anisotropic deformation (4). Emph% and fSAD% are used to quantify the emphysematous lung tissue destruction and the extent of small airway narrowing or closure, respectively (5). β_{tissue} indicates the proportion of tissue volume for detection of tissue destruction.

Supplementary Content 2: Abbreviation List of qCT Variables

The region where the qCT variable was measured was specified as the subscript of the variable, namely $\{\text{Variable}\}_{\{\text{Region}\}}$.

Regions:

LUL: Left upper lobe

LLL: Left lower lobe

RUL: Right upper lobe

RML: Right middle lobe

RLL: Right lower lobe

Total: Total lung

RMB: Right main bronchus

LMB: Left main bronchus

BronInt: Right intermediate bronchus

TriLLB: Trifurcation of left lower lobe

sLUL: Sub-grouped segmental airways of left upper lobe

sLLL: Sub-grouped segmental airways of left lower lobe

sRUL: Sub-grouped segmental airways of right upper lobe

sRML: Sub-grouped segmental airways of right middle lobe

sRLL: Sub-grouped segmental airways of right lower lobe

Structural Variables:

θ: Bifurcation angles between the children branches of trachea and RMB

Cr: Airway circularity

WT^*: Normalized airway wall thickness

D_h^*: Normalized airway hydraulic diameter

Functional Variables:

ΔV_{air}^{F}: Fractional air volume change

J: Determinant of Jacobian matrix

ADI: Anisotropic deformation index

fSAD%: Fraction-based small airways disease

Emph%: Fraction-based emphysema

β_{tissue}: Tissue fraction at TLC

$\Delta V_{air,UML}^{F}$: The ratio of the air-volume change of the upper lobes to the air-volume change of the middle and lower lobes combined

Supplementary Content 3: A Post-hoc Study

A post-hoc study was conducted to establish the causal relationship between fSAD and heterogeneity of lung ventilation. Cross-lagged panel analysis, which is commonly used to infer the direction and strength of a relationship between two variables repeatedly measured at different time points, was employed (6). The two imaging variables of $fSAD\%_{Total}$ and CV_{Total} at V1 and V2 were chosen for analysis since they were strongly correlated at all visits. The type one error rate (α) was set at 0.05. As demonstrated in **Figure S2**, two synchronous correlations ($r_{fSAD1,CV1}$ and $r_{fSAD2,CV2}$) and two stability correlations ($r_{CV1,CV2}$ and $r_{fSAD1,fSAD2}$) were both significantly greater than zero, indicating that the assumptions of synchronicity and stationarity were not violated. Regarding the cross-lagged correlations ($r_{fSAD1,CV2}$ and $r_{CV1,fSAD2}$), $r_{fSAD1,CV2}$ was significantly greater than zero while $r_{CV1,fSAD2}$ was not, suggesting that fSAD is the cause of heterogeneity of lung ventilation. Note that the cross-lagged correlations were partial correlations which the contributions of the stability correlations had been partialled out.

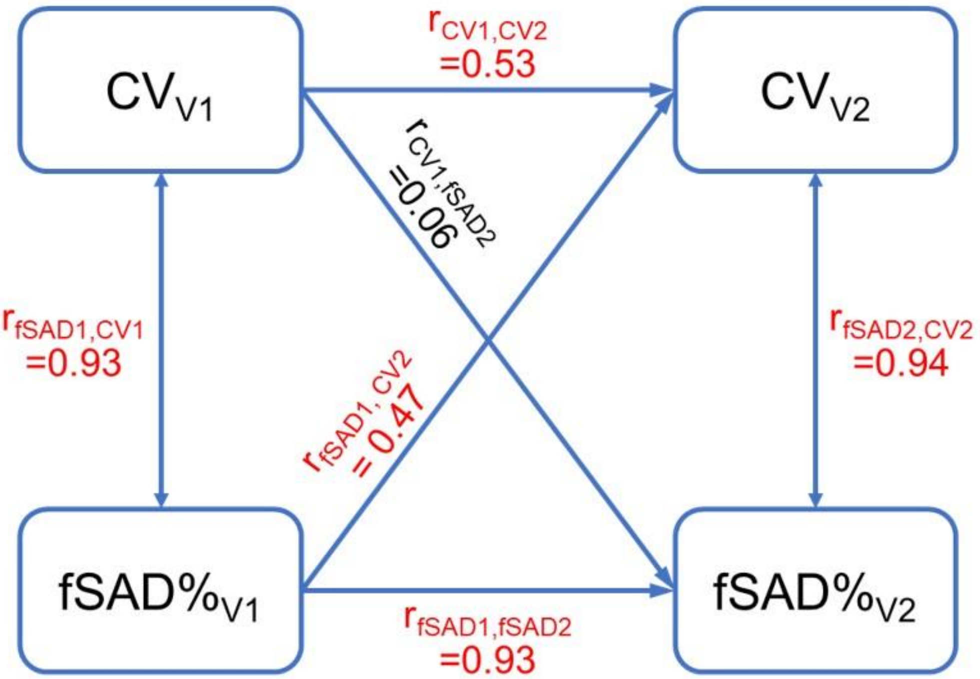

Figure S2. The cross-lagged panel analysis estimated a total of six correlations. The correlations colored in red were significantly greater than zero. The result that $r_{fSAD1,CV2}$ was greater than zero suggested that fSAD cause the heterogeneity of lung ventilation.

Supplementary Reference

1. Onoe R, Yamashiro T, Handa H, Azagami S, Matsuoka S, Inoue T, et al. 3D-measurement of tracheobronchial angles on inspiratory and expiratory chest CT in COPD: Respiratory changes and correlation with airflow limitation. Int J COPD. 2018;13:2399–407.
2. Vasilescu DM, Martinez FJ, Marchetti N, Galbán CJ, Hatt C, Meldrum CA, et al. Noninvasive imaging biomarker identifies small airway damage in severe chronic obstructive pulmonary disease. Am J Respir Crit Care Med. 2019;200(5):575–81.
3. Choi S, Hoffman EA, Wenzel SE, Castro M, Fain SB, Jarjour NN, et al. Quantitative assessment of multiscale structural and functional alterations in asthmatic populations. J Appl Physiol. 2015;118(10):1286–98.
4. Amelon R, Cao K, Ding K, Christensen GE, Reinhardt JM, Raghavan ML. Three-dimensional characterization of regional lung deformation. J Biomech [Internet]. 2011;44(13):2489–95. Available from: http://dx.doi.org/10.1016/j.jbiomech.2011.06.009
5. Galbán CJ, Han MK, Boes JL, Chughtai KA, Meyer CR, Johnson TD, et al. Computed tomography-based biomarker provides unique signature for diagnosis of COPD phenotypes and disease progression. Nat Med. 2012;18(11):1711–5.
6. Kenny DA. Cross-lagged panel correlation: A test for spuriousness. Psychol Bull [Internet]. 1975 Nov;82(6):887–903. Available from: papers://9813c380-4a5a-426e-ab3b-10e227534cf8/Paper/p540
